# Hair Regeneration Methods Using Cells Derived from Human Hair Follicles and Challenges to Overcome

**DOI:** 10.3390/cells14010007

**Published:** 2024-12-25

**Authors:** Ons Ben Hamida, Moon Kyu Kim, Young Kwan Sung, Min Kyu Kim, Mi Hee Kwack

**Affiliations:** 1 Department of Immunology, School of Medicine, Kyungpook National University, Daegu 41944, Republic of Korea; ons.benh94@gmail.com (O.B.H.); moonkim@knu.ac.kr (M.K.K.); ysung@knu.ac.kr (Y.K.S.); mq783@hanmail.net (M.K.K.); 2 Hair Transplantation Center, Kyungpook National University Hospital, Daegu 41944, Republic of Korea

**Keywords:** human hair follicle regeneration, dermal papilla cells, dermal sheath cells, epithelial stem cells

## Abstract

The hair follicle is a complex of mesenchymal and epithelial cells acquiring different properties and characteristics responsible for fulfilling its inductive and regenerative role. The epidermal and dermal crosstalk induces morphogenesis and maintains hair follicle cycling properties. The hair follicle is enriched with pluripotent stem cells, where dermal papilla (DP) cells and dermal sheath (DS) cells constitute the dermal compartment and the epithelial stem cells existing in the bulge region exert their regenerative role by mediating the epithelial–mesenchymal interaction (EMI). Many studies have developed and focused on various methods to optimize the EMI through in vivo and in vitro approaches for hair regeneration. The culturing of human hair mesenchymal cells resulted in the loss of trichogenicity and inductive properties of DP cells, limiting their potential application in de novo hair follicle generation in vivo. Epithelial stem cells derived from human hair follicles are challenging to isolate and culture, making it difficult to obtain enough cells for hair regeneration purposes. Mesenchymal stem cells and epithelial stem cells derived from human hair follicles lose their ability to form hair follicles during culture, limiting the study of hair follicle formation in vivo. Therefore, many attempts and methods have been developed to overcome these limitations. Here, we review the possible and necessary cell methods and techniques used for human hair follicle regeneration and the restoration of hair follicle cell inductivity in culture.

## 1. Introduction

The hair follicle (HF) is a “mini-organ” in which hair follicle stem cells and progenitor cells coexist [1]. Therefore, HFs can serve as a reservoir of pluripotent stem cells capable of regenerating all skin lineages [2,3]. The HF is formed by epithelial–mesenchymal interaction (EMI) between the stem cells residing in the bulge region, namely epithelial stem cells (Epi-SCs), and the stem cells present in mesenchymal components (dermal papilla (DP) and dermal sheath (DS) cells). Therefore, the EMI of both cells is required for hair follicle morphogenesis and regeneration. Injecting a combination of Epi-SCs and DP cells into immunodeficient mice has the potential to generate new hair follicles with a proper multi-layered stratified epidermis that includes structures resembling hair follicles [4]. A combination of epidermal cells and mesenchymal cells isolated from mice can form hair follicles with hair cycling properties through EMI. The postnatal hair follicle (HF) undergoes a well-defined cyclical process consisting of growth (anagen) leading to rapid cell proliferation, regression (catagen), which is a transitional period characterized by apoptosis-driven regression, and rest (telogen) [1].

However, human-derived dermal papilla (DP) cells and epidermal stem cells (Epi-SCs) face challenges in forming hair follicles due to the loss of genes involved in follicle formation during the culture process and the difficulty in obtaining a sufficient number of cells with trichogenetic capacity [5,6]. Therefore, numerous attempts have been made to restore the expression of genes involved in hair follicle formation [7,8,9]. Limited research has been conducted on the formation of human hair follicles using a combination of human-derived Epi-SCs and DP cells [10,11]. Consequently, restoring the hair-follicle-inducing ability of human DP cells requires a complex process of adjusting or modifying cell culture conditions [8,12,13,14]. For human Epi-SCs, culturing methods require a sufficient number of cells for hair regeneration experiments [10,15]. In this review, various techniques for maintaining or restoring hair-follicle-inducing ability and for hair follicle regeneration are being evaluated (Table 1).

## 2. Cells Required for Human Hair Follicle Formation

### 2.1. Dermal Papilla Cells and Dermal Sheath Cells

The growth and cycling of the hair follicle are controlled by a cluster of specialized mesenchymal cells termed as dermal papilla cells (DP cells). They are derived from the dermal condensate and are fundamental for hair follicle development upon interaction with Epi-SCs [37,38]. Various methods have been utilized to isolate DP cells from human hair and murine whisker follicles [39,40] to investigate their de novo inductive characteristics after interaction [41,42] with the surrounding epidermal environment.

For regeneration research, primary dermal papilla (DP) cells of hair follicles can be effectively isolated using fluorescence-activated cell sorting (FACS) with transgenic fluorescent proteins or through microdissection techniques. For example, GFP- or RFP-positive DP cells can be sorted by means of FACS from transgenic mice that express versican or Lef1 in the DP cells [43,44]. However, isolating DP cells from human hair follicles cannot be achieved using the same methods applied to mice [2]. Human DP cells are obtained from hair follicles sourced from patients undergoing hair transplantations. However, since the supply of human scalp samples is limited, the most commonly adopted method for cell expansion involves isolating and culturing DP cells for hair regeneration research. Another limitation exhibited by DP cells is that their culturing reduces their inductivity to generate hair follicles by passage [12,27,28]. Indeed, freshly isolated human DP cells and cultured DP cells differ significantly in their expression of key signaling pathways, such as WNT, BMP, and FGF, as shown in microarray analyses [8,12,25,28]. For this reason, researchers have explored various approaches to restoring the lost inductivity of DP cells in human hair follicles. Recent attempts have been made to restore the hair-regenerating ability of human dermal papilla (DP) cells once it has been lost. Various approaches have been explored, including the recovery of hair follicles’ regenerative ability through 3D cultures, restoration via the activation of the Wnt/β-catenin signaling pathway using GSK3β inhibitors, enhancement of hair follicles’ regenerative ability through exosome treatment, and the induction of human iPSCs by applying Yamanaka factors.

Markers such as versican [45,46,47], ALP [29,48,49,50], SOX2 [51,52], and CD133 [53] are known to be involved in hair follicle regeneration. Among these, CD133, a cell surface marker, is recognized as a DP-specific marker critical for hair follicle regeneration, particularly due to its expression in mouse DP cells during the anagen phase [53]. However, CD133 is exclusively expressed in mice and is not detected in human DP cells.

Dermal sheath (DS) cells, located on the outermost layer of the hair follicle, envelop the entire follicle. The role of DS cells has also been important in fueling and enhancing the inductivity of DP cells. The isolation and culture of DS cells from mouse vibrissa can lead to the formation of new hair follicles, demonstrating their regenerative potential [54,55,56]. Cultured DP cells exhibited a significant decrease in their inductivity due to a lack of aggregation and loss of potency in later culture passages [30,40]. Co-cultures of DP cells with DS cells demonstrated a promising increase in the proliferating function and self-renewal pattern of DP cells along with increasing their paracrine effects through interactions with epithelial cells. Various studies have discussed the important role of DS cells in murine and human hair follicle regeneration [31,33]. DS cells express α-smooth muscle actin (α-SMA), a marker absent in DP cells under in vivo conditions [55]. Interestingly, α-SMA is only expressed in cultured DP cells [40]. The DS has a contractile function that moves the DP towards the hair follicle bulge area during the catagen phase, facilitating crosstalk between the DP and Epi-SC [56]. To illustrate the positive effect of DS cells on DP cells and promoting the hair follicle’s entry into anagen [55], laser-based cell ablation of the mesenchyme forming DP and DS cells was performed, which showed no transition of the hair follicle into anagen, whereas the partial ablation of DS cells containing the hair follicle stem cells decreased the hair cycling progression. The DP and DS cells exhibited similarities in gene expression [57], and the expression of thrombin by DS cells stimulated the induction of differentiation of DP cells into myofibroblast-like cells in vitro [58]. Oliver et al. successfully generated rat whisker follicles by implanting DP cells at the base of inactivated non-cycling follicles [59]. They also confirmed that DP cells alone can generate a hair follicle, but the entire papilla is crucial for the induction of a cycling hair follicle, emphasizing the importance of the presence of DS cells and their interaction with DP cells [55,60]. Furthermore, by implanting DS cells isolated from a male donor into the arm of a genetically unrelated female recipient, Reynolds et al. confirmed the ability of DS cells to induce the growth of the hair follicle by stimulating DP cells [61]. The inductive effect of DSCs in the DS on DP cells was also confirmed in wound healing based on the observation that the cells exhibited similar developmental and anatomical behavior to that of ORS cells [62]. The hair follicle recycling and regenerating effect exerted by DS cells has also been observed in the epithelial compartment, specifically the ORS cell progenitors. The DS–ORS interaction results in giving rise to the matrix transit amplifying cells that engulf DP cells [63]. The in vitro expansion of lower DS (DSC) cells obtained from occipital scalp biopsies and subsequently injected into the human scalp has significantly increased total hair density and cumulative hair diameter at the injection site compared to a placebo after 6 to 9 months, although no further increase was observed 12 months post-injection [64]. This suggests that DSC cells induce the formation of new hair follicles throughout their interaction with Epi-SCs present in the human occipital region. By serving as a reservoir for DP cells replenishment, DS cells are efficient in inducing the regeneration and growth of hair follicles.

### 2.2. Epithelial Stem Cells

Cells with high colony-forming ability and label-retaining properties in the bulge area of the hair follicle are referred to as bulge stem cells, epithelial stem cells (Epi-SCs), or hair follicle stem cells (HFSCs) [65]. Hair follicle epithelial stem cells (Epi-SCs) are essential for the maintenance and renewal of the cycling hair follicle by inducing the keratinization of the hair shaft [66]. They represent one of the two major compartments responsible for the formation of a fully functional and cycling hair follicle, along with the mesenchymal cells of the follicular papilla [67]. Epi-SCs showed different expression patterns in mice and humans through gene expression and immunostaining. Keratin 15 is expressed in the bulge of both mice and human hair follicles [68,69,70]. CD200 is an Epi-SC marker expressed only in humans [69,70,71], compared to CD34, a mouse-specific Epi-SC [68,72,73]. It has been reported that bulge stem cells are required for hair follicle formation. Bulge stem cells were isolated from β-galactosidase-labeled vibrissae and implanted into the normal bulge region of a mouse whisker, which resulted in the generation of the entire hair shaft and follicular epithelium [74]. These studies confirmed the contribution of bulge stem cells in producing different hair follicle compartments [38], as well as their major involvement in anagen transition by interacting with DP cells [75]. In murine studies, the isolation of Epi-SCs [76,77] using the fluorescence-activated sorting or magnetic sorting of cells expressing α6 integrin and CD34 [78] or cells from YFP (yellow fluorescent protein) reporter mice [77] was helpful in investigating their inductive role in de novo hair follicle formation. In human neonatal foreskin, CD200 expressing Epi-SCs indicated an important role in establishing the hair-immune privilege, as well as a significant increase in colony formation in clonogenic assays when expanded in vitro [65]. In hair regeneration assays, the use of human-derived dermal and epidermal cells has not been efficient in cycling hair follicle formation because of the loss of the inductive abilities of stem cells when cultured in vitro. However, the combination of versican-GFP-derived DP cells from mice and neonatal human keratinocytes successfully generated a chimeric hair-follicle-like structure [47]. Another successful method for generating hair follicles and sebaceous glands has been reported, which involved combining mouse-derived dermal cells and human ectodermal precursor cells derived from iPSCs and injecting them into the fascia of immune-deficient mice [79].

**Figure 1:**
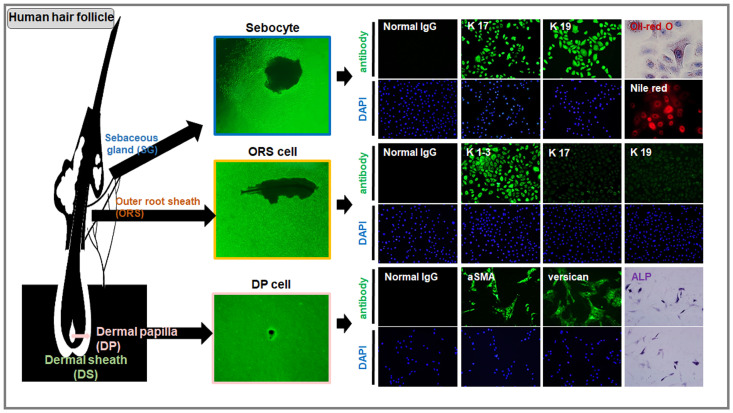
Morphology and markers of cells isolated and cultured from human hair follicles. These are the results of staining with various markers after isolating and culturing cells from human hair follicles. The image shows dermal papilla (DP) cells after two weeks of culture, expressing a-SMA, versican, and ALP in cultured DP cells (passage 1). Outer root sheath (ORS) cells were isolated and cultured from the ORS derived from the epithelium, and the image shows the cells on day 10. Keratin 1–3 were strongly expressed, while keratin 17 and 19 were not expressed. Additionally, sebocytes were isolated and cultured from human sebaceous glands (SG), with the image showing the cells on day 10. These sebocytes strongly expressed keratin 17 and keratin 19, and lipid secretion was observed through Oil Red O and Nile Red staining, adapted with permission from Ref. [80].

As shown in these results, Epi-SCs were derived from humans, but dermal cells were isolated from mice. No methods of hair formation using human-derived dermal cells and human-derived epidermal cells have been reported yet. The formation of a functional and cycling hair follicle using only human-derived dermal and epidermal cells is still challenging. Unfortunately, the current method of isolating bulge cells directly from human HFs is relatively impossible and the efficiency is too low, along with the loss of their inductive abilities in forming hair follicles when cultured in vitro (Figure 1).

## 3. Restoration of the Inductivity of Hair-Follicle-Derived Cells

### 3.1. Induced Pluripotent Stem Cells

Hair follicle stem cells exist in multiple lineages of human hair follicles. Their use in regeneration assays is rather difficult due to the limited number of isolation methods and low sample numbers. Therefore, several groups have induced human-induced pluripotent stem cells (hiPSCs) to produce similar biological properties to their primary state [17,18,81,82]. This also represents an alternative approach avoiding the use of HF tissue donors that might result in ethical issues when proceeding with regeneration procedures. The use of iPSCs is largely accessible due to their proliferating ability and pluripotent nature, meaning that only a limited number of cells are required. However, the long-term culture requirements and low success rate can present limitations to the use of iPSCs.

Reprogramming human pluripotent stem cells (hPSCs) into neural crest (NC) cells and plating them on fibronectin-coated plates resulted in the expression of NC cell-specific markers, viz, LNGFR, Sox10, and foxd3 [83]. Exposing NC cells to DP conditioned medium resulted in their conversion into versican, α-SMA-expressing DP-like cells in culture [83,84]. The transplantation of the mixture of iPSCs-derived DP cells with murine neonatal hair epithelial cells into immunodeficient mice resulted in hair follicle formation. The reprogramming of human hair follicle mesenchymal stem cells mediated by Yamanaka factors (oct4, sox2, c-Myc, and Klf4) was found to aid the generation of personalized iPSCs capable of forming teratomas exhibiting all three germ layers when delivered into the skin of immunodeficient mice [85]. A co-culture of iPSC-derived DP cells and epidermal cells was efficient in the formation of three-dimensional organoids capable of generating a cycling hair follicle [16]. The reprogramming of iPSCs into epithelial cells can also be achieved using retinoic acid and BMP4 treatment to inhibit the induction of NC cells [86]. IPSCs-derived EPCs were expressed specific epidermal progenitor markers, KRT8 and KRT14 [72]. iPSCs-derived EPCs co-cultured with human DP cells induced the upregulation of LEF1, BMP4, and ALP expression specific to DP cells. This alternative approach highlighted the potential of reprogramming somatic stem cells in vitro to generate autologous pluripotent stem cells capable of being differentiated into functional cells that aid hair follicle formation.

### 3.2. Treatment of Stem Cell-Derived Conditioned Medium and Exosome

A study addressing the role of hair follicle stem cells on mesenchymal stem cells and Epi-SCs in androgenetic alopecia (AGA), common male pattern baldness, and pathogenesis revealed the loss of their potential to differentiate into progenitor cells, resulting in the miniaturization of hair follicles [72]. The use of a stem-cell-derived medium for restoring the activity of DP cells represents a novel approach for stimulating the growth and regeneration of hair follicles. The DP cells of AGA hair follicles are characterized by a decrease in the number of their hair follicle stem cell progenitors [72]. To reverse the inhibition of the miniaturizing effect of AGA on the hair follicle, methods for activating DP cells [87] or bulge Epi-SCs [88] have been adopted. The use of a conditioned medium (CM) or the secretome containing cytokines, growth factors, and extracellular vesicles (EVs) secreted by cultured stem cells [89,90] was found to exert a positive impact on follicular cell growth and hair development. The use of CM derived from adipose-derived stem cells (ADSCs) increased the number of anagen hair follicles by stimulating the activation and differentiation of hair follicle stem cells through the activity of growth factors, IGF and VEGF, which is enhanced under hypoxic conditions [91,92,93]. Inamatsu et al. demonstrated the inductive capacity of cultured rat dermal papilla (DP) cells when treated with conditioned medium (CM) derived from sole skin keratinocytes [19]. Additionally, Qiao et al. and Inuo et al. showed that the trichogenicity of human DP cells was maintained when treated with CM from the keratinocytes of newborn foreskin and human facial skin, respectively [5,94]. Lee et al. further reported that CM from human outer root sheath keratinocyte cultures activates signaling pathways that support the maintenance of hair-inducing properties and enhance the trichogenic potential of dermal cells [20].

Exosomes are small extracellular vesicles (30–150 nm in diameter) formed within multivesicular bodies and released into the extracellular space via exocytosis. Their lipid bilayer structure protects their cargo, which includes growth factors, signaling molecules, microRNAs (miRNAs), and cytokines [95]. These vesicles can influence recipient cells by delivering bioactive molecules, making them effective mediators in regenerative medicine [95]. Therefore, exosomes isolated from various types of stem cells have been extensively studied for their roles in signaling pathways related to hair growth and hair regeneration [96]. Exosomes derived from human DP cells have been shown to promote the proliferation of outer root sheath (ORS) cells and DP cells, enhance the length growth of human hair follicles, and delay the transition to the catagen stage [21,97,98]. Furthermore, Kwack et al. demonstrated that exosomes derived from human DP cells cultured as spheres (3D DPC-EVs) triggered the transition from telogen to anagen and extended the duration of anagen [99]. Moreover, when 3D DPC-EV-treated spheres were co-implanted with fresh mouse epidermal cells in a hair reconstitution assay, the formation of new hair follicles was induced [21]. In a study involving androgenetic alopecia (AGA) patients, the application of adipose-derived mesenchymal stem cell (MSC) exosomes using a microneedle roller resulted in increased hair density and thickness [99]. In addition, exosomes derived from human or mouse bone marrow mesenchymal stem cells [100,101], human fibroblasts [102], human neural progenitor cells [103], and mouse vibrissa DP cells [104] have also been shown to induce the proliferation of dermal papilla (DP) cells and activate Wnt/β-catenin signaling pathways.

### 3.3. Treatment of Small Molecules or Inhibitors

The experimental implantation of dermal papilla (DP) cells has been shown to induce the formation and growth of hair follicles in mice [6], with the process being dependent on small molecules or inhibitors. To promote follicular regeneration and growth by activating Wnt signaling, approaches include overexpressing Wnt3a [105], inhibiting GSK3-β using BIO (6-bromoindirubin-3′-oxime) [22] or CHIR99021 [23], or activating Wnt/β-catenin signaling with KY19382 [24]. The Kishimoto team demonstrated that 0.5 µM BIO [22] or 3 µM CHIR99021 [23] promoted the proliferation of human dermal papilla (DP) cells and increased the expression of trichogenic markers such as ALP, LEF1, and Versican. Using a sandwich model method with a murine epidermal cell fraction for xenografting or a human reconstituted skin assay for xenografting, they successfully induced hair follicle formation [22,23]. Ryu et al., reported that when the synthesized analog Wnt activator, 5 µM KY19382, was applied to human dermal papilla (DP) cells, it promoted human hair growth. Furthermore, when mouse dermal cells and epidermal cells treated with KY19382 were combined and subjected to a patch assay, hair follicle formation was successfully induced [24]. BMP6 is strongly expressed in dermal papilla (DP) cells [106], and the treatment of cultured DP cells with BMP6 increases the expression of trichogenes [25,106]. When mouse DP cells treated with 400 ng/mL BMP6 were mixed with epidermal cells and subjected to a chamber assay, hair follicle formation was successfully induced [25]. Beads coated with 100 ng/μL FGF7 are involved in initiating a new hair cycle [107]. Isolated and cultured dermal papilla (DP) cells in human hair follicles (HF) exhibit lower levels of WNT, BMP, and FGF-related signals compared to freshly isolated DP cells [12,25]. For this reason, treatment with the combination of FGF2, BMP2, and BIO in cultured human DP cells could induce the generation of DP-like cells from fetus- or adult-foreskin-derived fibroblasts [12,25]. The pharmacological inhibition of the Janus kinase (JAK)–signal transducer and activator of transcription (STAT) pathway promotes rapid hair regrowth in alopecia areata (AA) in both mice and humans [26]. The inhibition of JAK signaling by ruxolitinib (400 nM) or tofacitinib (400 nM) initiates the hair cycle in normal mice and promotes the growth of hair follicles in humans. Tofacitinib-treated human DP spheres combined with neonatal mouse keratinocytes promote hair follicle formation [26].

### 3.4. Three-Dimensional (3D) Cultures

The morphology, characteristics, and behavior of DP cells have been extensively investigated in vitro [8,108,109]. The dermal papilla (DP) plays a crucial role in hair regeneration and cycling by expressing hair growth factors and DP-specific genes that are essential for producing extracellular matrix (ECM) components, including laminin, fibronectin, and versican in vivo [6]. This expression leads to the dermal papilla’s strong self-aggregating properties [110,111,112]. Two-dimensional cultures of DP cells result in a decrease in their inductive potency, explained by the loss of activity of their anagen-related genes such as versican [46] and ALP [49] due to alterations in the extracellular matrix in high passages [8]. This change in gene expression inhibits the ability of the cells to aggregate, thus disrupting the growth and cycling properties of hair follicles. To restore and sustain gene expression, 3D culturing techniques have been employed, with successful outcomes reported in reversing the loss of activity in various genes [8,27,40]. The injection of 3D-cultured DP aggregates into immunodeficient mice has resulted in de novo hair formation [12,28,30]. Methods such as the hanging drop technique utilizing gravity [28], culturing cells on low cell-binding plates [30,50], three-dimensional cultures using self-assembly on poly(ethylene-co-vinyl alcohol, EVAL) membranes [40], and culturing on polyvinyl alcohol (PVA)-coated 96-well PCR plates have all been shown to enhance the aggregation of human DP cells, thereby promoting hair formation [31]. Three-dimensional spheroid DP cells play a role in hair follicle formation by structurally transforming high-passaged 2D-cultured DP cells into a form that expresses genes similarly to those in primary DP cells capable of generating hair. However, the aggregation of DP cells alone is insufficient to fully restore the hair-inductive properties lost in 2D culture. To achieve a complete hair structure, a variety of cells, including endothelial cells, DS cells, and epithelial cells, must coexist, as observed in the DP of hair follicles. Injecting a combination of human DP spheroids and neonatal mouse epidermal cells in the back of nude mouse was found to result in de novo hair generation [21,28,29,31,32]. By adapting the 3D culture technique, the hair follicle microenvironment has been re-established, exhibiting follicle-specific phenotypes in vivo [40].

### 3.5. Biomimetic Development Approach

Successful cases of de novo hair follicle neogenesis when rodent [113] or human intact DP cells [114] were transplanted into recipient mouse epithelium have been reported, emphasizing the DP reprogramming ability of the epidermis into a follicular fate. Using self-aggregating DP cells cultured under 3D conditions has also resulted in the mimicking of cell condensation behavior [12]. Nevertheless, adopting these approaches might also present various limitations in promoting hair follicle morphogenesis. The partial restoration of the intact DP signature’s gene expression or interference with the epidermal–mesenchymal interactions can also occur due to the dissociation of spheroids in culture [33]. To overcome these limitations and recreate a favorable microenvironment for the hair follicle enabling the recapitulation of its 3D conformation and stimulating its generation, an innovative biomimetic method was implemented using 3D printing technology. Three-dimensional printing technology induces the epidermal–mesenchymal interaction by controlling the aggregation of DP spheroids into the extracellular matrix, resulting in the formation of a bioengineered hair follicle in vitro that enhances its survival when grafted in mice [33].

### 3.6. Skin Organoids

The establishment of organoid technology as an alternative for hair follicle regeneration has received extensive attention for the purposes of rescuing and recapitulating the key traits of hair follicles in vivo, reducing animal use, and also evaluating the efficacy of therapeutic approaches [34,35,115]. One protocol explains the achievement of organoids using human pluripotent stem cells (hPSCs). Further, hPSCs were cultured in a U-shaped 96-well plate to induce their aggregation. Next, the cell aggregates were treated with TGF-β inhibitor, BMP4, and bFGF to stimulate their differentiation and development of the dermal layer. A combined treatment with FGF2 and BMP4 inhibitor (LDN) was also performed to induce the epidermal layer of the organoid [34]. Floating cultures and the Matrigel-embedding method were also adapted for organoid cultures [116].

### 3.7. Use of Biomaterials as Hair-Follicle Substitutes

**Figure 2:**
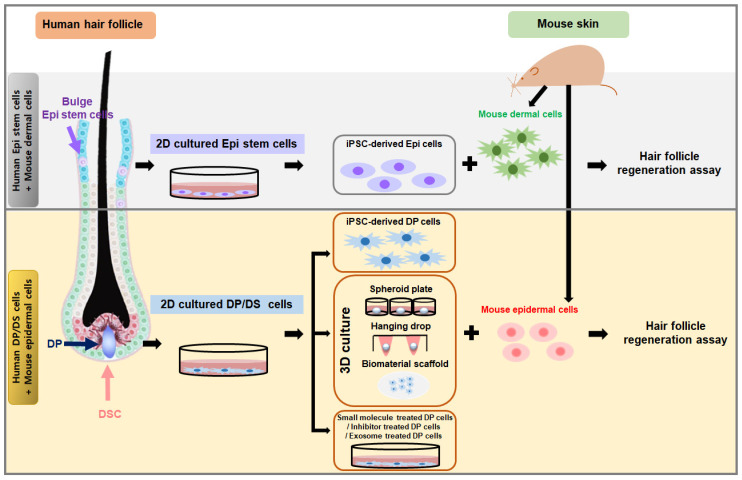
Method to restore the trichogenicity of cultured human dermal papilla (DP) cells or epithelial stem cells (Epi stem cells). Cultured human epithelial stem cells (Epi stem cells) are induced into iPSCs, mixed with mouse dermal cells, and utilized in hair regeneration assays to promote hair follicle formation. Cultured dermal papilla cells (DP cells) recover their hair follicle regenerative ability through iPSC induction, 3D cultures, or treatment with small molecules, inhibitors, or exosomes. These treated DP cells are then combined with mouse epidermal cells and employed in hair regeneration assays to induce hair follicle formation.

Biomaterials have been used for tissue regeneration and niche stimulation by establishing a microenvironment that enables cell interactions [9,117]. Using a mixture of biomaterials such as 3D hydrogels, ECM hydrogels [63], synthesized ECM [118], poly(ethylene-co-vinyl alcohol) [48], and scaffolds (collagen–chitosan scaffold) [36] with DP cells and epidermal cells was found to induce hair follicle regeneration. The self-aggregating behavior of these cells is gradually lost in vitro, with rapid disintegration occurring on a 2D culture surface [119] or within a collagen matrix [33,120]. This loss of aggregation is linked to the origin of DP cells, as demonstrated in a study using collagen-glycosaminoglycan hydrogels seeded with murine DP (mDP) or human DP (hDP) cells to create skin substitutes [121]. The positive population of factors involved in hair follicle formation, such as ALP, is high (Figure 2).

## 4. Hair Regeneration Assay

### 4.1. Patch Assay

The patch assay is a hair follicle regeneration method performed to reconstitute the mature hair follicle by manipulating cells in culture to facilitate the study of their trichogenicity. This assay overcomes the shortcomings of the chamber assay following the protocol established by Zhen et al. [122], which achieved the formation of mature hair follicle in the dermis by injecting freshly isolated epithelial and mesenchymal cells from neonatal mice into the hypodermis of a recipient mouse. The number of cells used was relatively low compared with that used in other assays such as the chamber assay, with only 10^6^ cells being injected, and different treatment conditions were applied in the same animal model, resulting in the appearance of hair follicles within 2 weeks of injection. The generation of the hair follicle requires the presence of both epithelial and dermal cells [123,124]. The injection of only epidermal or dermal cells results instead in the appearance of cysts or fibrotic stroma [33,35]. The patch assay was also conducted using human-derived DP spheroids cultured in a 96-well low-binding plate that were injected with neonatal mouse epidermal cells in the dorsal skin of nude mice [21,28,29,32]. Huang et al. developed a PVA-coated PCR tube method for the mass production of human and murine DP spheroids with a widely controllable size and cell number [31]. They investigated the effect of DP spheroid size on the induction efficiency and thickness of hair regenerated by mixing formed spheres and mouse epidermal cells [31]. Zhang et al. formed DP spheres on chitosan/PVA nanofibers and regenerated new hair follicles by combining them with mouse epidermal cells [118].

### 4.2. Chamber Assay

**Figure 3:**
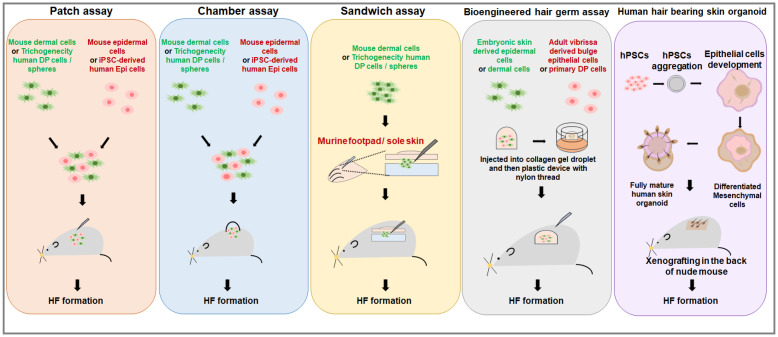
Currently reported methods for hair regeneration using cultured cells. This describes the models used to evaluate hair regeneration with isolated cells reported to date. Patch Assay: In this model, epidermal and dermal cells are mixed and injected subcutaneously into a nude mouse. Hair follicle formation can be observed two weeks later. Hair follicles do not form when using mouse dermal or epidermal cells alone, but mixing both types of cells results in hair follicle formation after two weeks. Chamber Assay: A chamber is inserted into a cut in the skin of a nude mouse, and a mixture of mouse dermal and epidermal cells is added to the chamber. Two weeks later, the chamber is removed, revealing a layer formed by the two cell types. By four weeks, hair follicle formation can be observed. Sandwich Assay: Dermal cells are placed between the epidermis and dermis of the footpad or sole skin, and the tissue is xenografted into another mouse. Hair follicle formation potential is then evaluated. Hair Germ Assay: Dermal and epidermal cells are mixed into a collagen gel drop, which is then injected into a nude mouse. Hair follicle formation is observed. Bearding Skin Organoid: Human pluripotent stem cells (hPSCs) are induced into iPSCs and treated with various chemicals to induce hair follicles in vitro.

The chamber assay model was established [42,125] to enable hair follicle reconstitution in nude mice. In this process, a combination of 10^7^ mouse epidermal and dermal cells is introduced into a silicone chamber inserted in the back skin of a surgically wounded nude mouse. The chamber is typically removed 1 to 2 weeks after transplantation, allowing the epithelial–mesenchymal interactions (EMI) between the two cell types to begin, leading to the hair establishment of a cellular layer on the skin surface. By 4 weeks post-injection, the outward growth of hair follicles can be observed. However, this assay can have disadvantages due to the large number of cells required for testing [126], and only one condition per mouse can be tested, leading to a lack of variety in testing conditions. Xiao et al. proposed the mini chamber assay, which allows for testing several types of experiments on a single nude mouse using a small number of cells [127]. Zhang et al. conducted a chamber assay by combining human DP spheres formed using chitosan/PVA with newborn mouse epidermal cells, demonstrating the regeneration of hair follicles outside the skin [118]. Xiao et al. used a PRP (platelet-rich plasma) scaffold to form DP spheres that showed increased levels of ALP and versican [128]. By using this method, a mixture of mouse 3D DP cells and mouse epidermal cells successfully induced hair follicles. However, when human DP spheres formed with the PRP scaffold were combined with human neonatal foreskin for a chamber assay, hair follicle induction did not occur. To date, there have been no reports of successful hair follicle regeneration using DP cells and epidermal cells derived from humans [128,129]. Therefore, to evaluate the hair follicle formation ability of human DP cells, they can be mixed with mouse-derived epidermal cells to assess their hair follicle regenerative potential. Similarly, to evaluate the hair follicle formation ability of human epidermal cells, their regenerative potential can be assessed by mixing them with mouse dermal cells (Figure 3).

### 4.3. Sandwich Assay

Reynolds et al. developed the sandwich assay based on the implantation of isolated DP aggregates from a donor adult rat pelage hair follicle inserted in between the epidermis and dermis of a follicular footpad. The combined tissue prepared above was then grafted into the subcutaneous area of the mouse [130]. This technique emphasized the important role of DP cell aggregates in inducing hair follicle neogenesis and the participation of epidermal cells derived from the non-follicular footpad characterized by embryonic-like behavior involved in the epithelial–mesenchymal interaction signals required for hair follicle formation. Higgins et al. obtained human foreskin from a children’s hospital, separated the epidermis and dermis using dispase treatment, and placed a DP sphere, formed via the hanging drop method, between the two layers. The combined skin was then grafted onto SCID (severe combined immunodeficiency) mice. After 3 weeks, hair formation was observed, resulting from the interaction between the human-derived DP aggregate and the human foreskin epidermis [12]. Hair follicles were successfully generated by aggregating human-derived DP cells through the hanging drop method and injecting them into human foreskin. After 3 weeks, hair formation was confirmed through the interaction between the DP aggregates and the epidermis [12].

### 4.4. Bioengineered Organ Germ Assay

Tsuji et al. established this method to develop alternative, correct, and fully functional organs to enable the restoration of damaged or unrepairable organs after injuries or diseases [131]. This approach is based on generating bioengineered ectodermal organs such as the tooth or hair follicle capable of developing in vitro and in vivo [131]. The bioengineered hair follicle germ assay has proven to be an effective approach for creating a complete organ by isolating embryonic skin epidermal and dermal cells and injecting them into a collagen gel droplet at a high cell density. These bioengineered hair germs are then placed on a cell culture insert with a pore size of 0.4 µm and incubated at 37 °C for two days. To facilitate epithelial connections between the host skin and the bioengineered hair follicle, a nylon thread is passed through the epithelial and mesenchymal portions of the bioengineered hair germ. Following intradermal implantation, these bioengineered follicles can integrate with the host dermal epithelium, replicating the stem cell niche and supporting hair cycling. Additionally, the human bioengineered hair follicle germ, composed of dissociated epithelial cells from the bulge region and intact dermal papilla (DP) cells derived from the scalp hair follicles of a patient with androgenetic alopecia, produced a pigmented hair shaft at the transplantation site. In the permanent area, they autonomously establish connections with nerves and the arrector pili muscle, inducing piloerection [131,132].

### 4.5. Hair-Bearing Human Skin Generation

The generation of human skin and the reconstruction of appendages in culture has been challenging to achieve as an alternative approach to minimize the usage of mouse models designated to investigating skin disorders in vivo. Due to the differences exhibited by mouse skin compared to human skin in wound healing and translational research [133], and the limitations shown in human equivalents in mimicking complete human skin models [134,135], a new approach based on generating hair-bearing skin tissue from a homogenous population of human pluripotent stem cells (hPSCs) in 3D cultures in vitro has been established [34,35]. Based on the previously reported study of the formation of appendage-bearing skin organoids from differentiated epidermal and dermal cells originating from mouse pluripotent stem cells [17], Lee et al. aimed to co-induce surface ectodermal and mesenchymal cells derived from hPSCs by exposing the aggregated stem cells to different culturing media enriched with differentiation factors, BMP4 and TGFβ inhibitors (SB431542), for epidermal induction [35]. Conversely, the use of a BMP inhibitor (LDN) combined with FGF induced the generation of a cranial neural crest (CNC)-like structure that promotes the development of mesenchymal cells [12,25,34]. The incubation of cranial epithelial cells and neural crest cells within a spheric cell aggregates resulted in the observation of a formed cyst-like organoid exhibiting a stratified epidermis and dermis, pigmented hair follicles, sebaceous glands, Merkel cells, and sensory neurons. Single-cell RNA sequencing highlighted the composition of skin organoids and its equivalence to the human skin of fetal tissue during the second trimester (18 weeks) of gestation. Further investigations showed that the xenografting of skin organoids into the back of nude mice forms planar hair-bearing skin. This approach proved the efficiency of skin organoids to be self-assembled while maintaining their characteristics in vitro as well as in vivo, providing new insights into skin regeneration and disease modeling.

## 5. Conclusions

The de novo formation of thick and cycling hair follicles has been challenging in terms of efficiency and reproducibility using human-derived cells. The various approaches and techniques adapted in vitro and in vivo developed for follicular regeneration have shifted their focus to generating and stabilizing an adequate microenvironment that enables hair follicle formation to be applied in clinical practice. The majority of experimental approaches to induce cellular interactions for hair follicle neogenesis have been restricted to rodents, and their application to humans has encountered various limitations. In fact, the study of human medical conditions such as AGA using animal models to obtain insights on the pattern development and to reverse its miniaturizing effect might encounter some challenges in terms of specificity and taxonomic differences. Therefore, developing a more universal approach such as cell-based therapy that focuses on the reprogramming of stem cells or controlling the cellular growth conditions to stimulate establishment of the microenvironment required for cellular interaction and growth could be promising in terms of efficiency and reproducibility.

It is well known by now that human- but not rodent-derived hair follicle DP cells, the indispensable regulators of hair formation and the key factor for the growth and survival of surrounding epithelial cells, are susceptible to losing their inductive potential in culture. Stimulating the inductivity of DP cells in culture was achieved by co-cultures with DS cells in the presence of stimulating factors such as Wnt, BMP, and FGF, resulting in their aggregation and the de novo formation of hair follicles when injected into nude mice. The reprogramming of mesenchyme-derived embryonic stem cells into iPSCs that exert DP-like characteristics has also shown positive results when translated into animal models via the patch, chamber, sandwich, and hair germ assays. The transition of DP cells in in vitro cultures into 3D spheroids also exhibited a positive response when used in in vivo approaches; however, it has also encountered experimental limitations in terms of cell number and cell environmental control. The development of organoids and the establishment of the physiological environment for cell transplants can also encounter limitations because of their different nature compared to normal tissue, which requires the presence of vascularization, immune cells, and connective tissues. The development of biomaterials and tissue engineering techniques has also been successful in hair follicle neogenesis under effectively controlled and stable internal and external conditions. The transplantation of bioengineered hair germs demonstrated an ability to install a vascularized environment, allowing the development of donor cell–host epithelium interaction irrespective of the placement depth. Hair-bearing human skin regeneration assays represent an efficient approach to inducing hair follicle formation by exposing human pluripotent stem cells to different culturing media, resulting in their differentiation into ectodermal and mesenchymal cells. The incubated cells form a sphere aggregate that results in the generation of hair follicles upon xenografting in the back skin of nude mouse. All of these attempts at mimicking the structure and behavior of a HF in its original tissue might result in achieving a fully functional and cycled hair follicle when combining different techniques, starting by expanding cells in culture and stimulating their aggregation and trichogenic activity to be subsequently transferred to patients with alopecia as a regenerative form of treatment. These of adopted methodologies have exhibited successful results when performed in vivo and delivered in a mouse model. They have also demonstrated promising hair-growth-inductive results when cell-based therapy was used as a form of regenerative medicine approach. There is currently a lack of research on whether hair follicles formed using cells undergo a hair cycle and how long these follicles can be maintained. Further studies are also required to determine whether hair follicles formed using human-derived dermal papilla (DP) cells resemble natural human hair follicles. Nevertheless, efficient cell and drug delivery methods, increasing hair follicle density, thickness, and orientation, and maintaining cycling properties in patients are still challenging tasks to achieve, which places the focus on developing potential techniques to enhance these limitations in clinical applications.

For patients with hair loss, treatment typically involves medications such as minoxidil or finasteride or hair transplantation. In the case of medication, discontinuing the drug often results in the recurrence of hair follicle loss. For hair transplantation, follicles from the patient’s occipital area are transplanted to the frontal area, but this method faces challenges when the number of transplantable follicles is insufficient. As an alternative, cell therapy offers a promising solution, where the patient’s own cells are cultured and expanded for transplantation onto their scalp. To make this approach viable, it is crucial to establish definitive conditions under which human DP cells can form hair follicles. Moreover, developing methods to induce hair follicle formation through the combination of human DP cells and epithelial stem cells is essential. As mentioned above, leveraging various strategies and techniques could help to address the challenges faced by patients with hair loss.

## Figures and Tables

**Table 1:** Overview of hair regenerative techniques using isolated and cultured hair-follicle-derived cells.

	Cells Utilized in the Regeneration Assay	Hair Regeneration Assay	Reference Number
1. Induced pluripotent stem cells (iPSC)	Co-cultured of human iPSC -derived DP cell and epidermal cells	Hair Bearin assay	[16]
	human iPSC-derived EpiSCs and mouse dermal cells	Chambe and Patch assy	[17]
	Co-culture of hiDPSCs with hkeratinocytes	Bioengineered hiar germ assay	[18]
2. Treatment of stem cell-derived conditioned medium and exosome	Keratinocyte condicition medium treated vibrisa DP cells	Sandwich assay	[19]
	Human keratinocyte condicition medium treated mouse dermal cell and mouse epiderma cell	Patch assay and Chamber assay	[20]
	Exosomes derived from human DP cells treated human DP cells and mouse epidermal cells	Patch assay	[21]
3. Treatment of small molecules or inhibitors	BMP 2, bFGF, BIO treated human DP cells	Sandwich assay	[8]
	GSK-3b inhibitor BIO treated human DP cells and mouse epidermal cells	Sandwich assay	[22]
	Wnt activator CHIR99021 treated human DP spheroids and mouse epidermal cells	Sandwich assay	[23]
	Wnt activator KY19382 treated mouse dermal and mouse epidermal cells	Patch assay	[24]
	BMP6 treated mouse DP cells and mouse keratinocytes	Chamber assay	[25]
	Jak inhibitor, Ruxolitinib or Tofacitinib treated human DP speroid and mouse epidermal cells	Patch assay	[26]
4. Three-dimensional (3D) culure	Human DP spheroids and human foreskin	Sandwich assay	[12]
	Mouse vibria DP spheroids and mouse peidermal cells	Patch assay	[27]
	Human DP spheroids and mouse epidermal cells	Patch assay	[26,28,29,30,31,32]
5. Biomimetric development approach	Human DP cell and keratinocyte using 3D printed molde	Skin graft assay	[33]
6. Skin organoids	Human embryonic stem cells	Skin graft assay	[34,35]
7. Use of biomaterials as hair follicle substitutes	Mouse DP cell using poly(ethylene-co-vinyl alcohol) (EVAL) membranes and mouse epidermal cell	Patch assay	[30]
	Mouse DP cell using collagen chitosan scaffold nd mouse epidermal cell	Chamber assay	[36]

## Data Availability

The data presented in this study are available in this article.
